# Substance use and sexual orientation among adolescents: Differences by age group and sex in the 2023 National Survey of Drug Use and Health

**DOI:** 10.1111/ajad.70087

**Published:** 2025-09-14

**Authors:** Timothy J. Grigsby, Rachel Hoopsick, Dylan Barker, Elise Devier, Amber Amis, R. Andrew Yockey

**Affiliations:** ^1^ Department of Social and Behavioral Health University of Nevada, Las Vegas Las Vegas Nevada USA; ^2^ Department of Health and Kinesiology University of Illinois Urbana‐Champaign Champaign Illinois USA; ^3^ Department of Public Health University of Mississippi Oxford Mississippi USA

## Abstract

**Background and Objectives:**

Adolescent substance use is a critical public health concern with significant long‐term consequences, yet research on disparities by sexual orientation remains limited. Despite the unique stressors faced by sexual minority youth—particularly bisexual individuals—that increase their risk for substance use, most studies investigate disparities among older adolescents and adults. Using recent national data, this study aims to fill this gap and inform targeted prevention and intervention efforts for sexual minority youth.

**Methods:**

We leveraged data from the 2023 National Survey on Drug Use and Health from youth aged 12–17 (*N* = 10,361). Using weighted logistic regression models, we examined the separate relationships between sexual orientation and past‐year substance use (i.e., tobacco, marijuana, and alcohol) stratified by age and sex assigned at birth.

**Results:**

Among adolescents, 4.73% reported tobacco use, 11.2% reported marijuana use, and 17.2% reported alcohol use. Logistic regression models indicated that, compared to heterosexual youth, bisexual youth had higher odds of using tobacco (OR = 2.00, 95% CI = 1.37–2.90), marijuana (OR = 1.85, 95% CI = 1.43–2.40), and alcohol (OR = 1.32, 95% CI = 1.02–1.69), while gay/lesbian youth had lower odds of tobacco use (OR = 0.47, 95% CI = 0.24–0.94), but higher odds of alcohol (OR = 1.60, 95% CI = 1.06–2.44). Notable differences were observed across sex and age.

**Conclusions and Scientific Significance:**

Findings from the current study highlight significant disparities in substance use among youth based on sexual orientation, particularly among bisexual adolescents. We recommend that prevention and intervention efforts incorporate age‐specific and gender‐sensitive approaches to address the unique stressors faced by sexual minority youth.

## INTRODUCTION

Substance use during adolescence is a critical public health concern due to its potential for immediate and long‐term health consequences, including but not limited to the development of substance use disorders, mental health comorbidities, and adverse social outcomes.^1^, ^2^ While previous research has extensively documented disparities in substance use by sociodemographic factors such as race, socioeconomic status, and geographic location,^3^ less is known about how substance use behaviors vary by sexual orientation among youth.

Existing literature suggests that sexual minority individuals experience unique distal and proximal stressors which contribute to increased risk for substance use.^4^, ^5^, ^6^ Distal stressors refer to external experiences such as discrimination and bullying, while proximal stressors involve internal processes such as internalized stigma or concealment of identity. Together, these stressors contribute to cumulative minority stress and adverse health outcomes. Most research on sexual orientation and substance use has focused on adults or older adolescents, leaving a gap in understanding substance use disparities among younger populations.^7^, ^8^ The 2023 National Survey on Drug Use and Health (NSDUH) is the first year to include sexual orientation measures for youth ages 12–17, offering a novel opportunity to examine these disparities among early adolescents. Although other large‐scale surveys, such as the Youth Risk Behavior Survey (YRBS), have previously included these measures, most recent findings remain limited to older datasets. For example, analyses of the 2015 YRBS found that lesbian, gay, bisexual, and questioning youth reported significantly higher rates of substance use compared to their heterosexual peers.^9^ The present study builds on this literature by leveraging newly available NSDUH data to provide updated estimates and insights into adolescent substance use disparities by sexual orientation.

Prior studies have consistently demonstrated higher rates of substance use among sexual minority youth compared to their heterosexual peers. A meta‐analysis found that lesbian, gay, and bisexual (LGB) youth were significantly more likely to use alcohol, tobacco, and marijuana than heterosexual youth.^8^ These disparities have been attributed to minority stress theory, which posits that sexual minority individuals face chronic stressors—such as discrimination and victimization—specifically because of their sexual identity, which in turn increase risk for maladaptive coping behaviors like substance use.^10^ Subsequent research has reinforced these findings and expanded our understanding of contributing factors. For example, a previous meta‐analysis^11^ linked minority stress processes to substance use disparities among sexual minority adolescents. Goldbach and Gibbs^12^ extended minority stress theory to better reflect the experiences of adolescents, emphasizing the developmental salience of family rejection, lack of autonomy, and constrained access to supportive peers or community spaces. Similarly, another meta‐analysis^13^ identified sexual minority status as a significant correlate of both substance use and risky sexual behavior in adolescence. Most recently, Lippold et al.^14^ emphasized the central role of parenting and familial context in shaping mental health and substance use outcomes among queer youth. Collectively, these studies suggest that substance use disparities are present for sexual minority adolescents with a multifaceted web of causation spanning social, familial, and developmental contexts. Further, evidence suggests that bisexual individuals, specifically, may experience greater substance use disparities compared to their gay and lesbian counterparts. This may be due, in part, to unique stressors such as bisexual invisibility (i.e., the marginalization of bisexual individuals within both heterosexual and gay/lesbian communities) and binegativity (i.e., hostility toward bisexual people, stereotypes that bisexuality is an unstable and illegitimate sexual orientation or bisexual individuals are sexually irresponsible) within heterosexual and LGBTQ+ communities.^15^, ^16^

Given that national indicators of adolescent substance use prevention are worsening,^17^ the inclusion of sexual orientation in the NSDUH for youth provides a unique opportunity to assess substance use disparities at a formative developmental stage. Understanding patterns of alcohol, marijuana, and tobacco use among youth ages 12–17 by sexual orientation can inform targeted prevention and intervention efforts tailored to sexual minority youth populations.

The present study uses data from the 2023 NSDUH to examine past‐year alcohol, marijuana, and tobacco use among youth by sexual orientation. Specifically, we address the following research questions: (1) Are there differences in past‐month use of tobacco, marijuana, and alcohol by sexual orientation among U.S. adolescents aged 12–17?, (2) Do these differences vary by sex assigned at birth (male/female)?, and (3) Do these differences vary by age group (12–13, 14–15, 16–17 years old)? This study extends previous prevalence work^18^ by examining differences across adolescent age groups and adjusts for additional covariates (e.g., race/ethnicity and major depressive episode) to estimate odds of past‐year substance use across sexual orientation. By leveraging nationally representative data, this study aims to address key gaps in the literature and provide insights into early disparities in substance use behaviors. Findings from this study have the potential to inform public health strategies that address substance use risk factors among sexual minority youth and promote health equity.

## METHODS

### Data source and procedure

A secondary analysis of the 2023 NSDUH was conducted. The NSDUH is a nationally representative cross‐sectional survey of noninstitutionalized individuals ages 12 and older conducted annually in the US assessing mental health, substance use, and behavioral health utilization.^19^ The NSDUH implements a complex sampling design to ensure adequate representation of individuals and oversampled youth and young adults. Details of the NSDUH survey methodology are described elsewhere.^20^ Importantly, 2023 was the first‐year sexual orientation was assessed by the NSDUH for youth ages 12–17 years old, and it is the most recent publicly available data set. NSDUH procedures were approved by the RTI International Institutional Review Board, and participants provided informed oral consent.

### Measures

Sexual orientation was assessed with the question, “Which one of the following do you consider yourself to be?” Response options included: 1 = “Heterosexual, that is, straight,” 2 = “Gay or lesbian,” 3 = “Bisexual,” 4 = “I use a different term,” 5 = “I am not sure about my sexual identity,” and 6 = “I do not know what this question is asking.” Respondents who selected “I use a different term” were categorized as “Other” in the current study and were prompted with the follow‐up question, “What term do you use to describe your sexual identity?” Respondents could then provide a written response of up to 50 characters. Adolescents who reported not knowing what this question was asking were excluded from our analyses.

Past‐year substance use was assessed with separate single‐item questions for alcohol, tobacco, and marijuana with response options of 0 = no and 1 = yes used for analysis.

Adolescents reported their age (categorized by NSDUH as 12–13, 14–15, 16–17), biological sex (male, female), race/ethnicity (Non‐Hispanic White, Non‐Hispanic African American, Non‐Hispanic American Indian/Alaskan Native, Non‐Hispanic Pacific Islander, Multiracial, and Hispanic), and past‐year major depressive episode (no/yes).

### Analysis plan

Prevalence estimates with confidence intervals were estimated for demographics of the sample (unweighted *n* = 10,361). Next, bivariate tests of substance use were estimated across all strata of sexual orientation. Finally, multivariable logistic regression models were built to determine the separate conditional associations of sexual orientation and past‐year tobacco use, alcohol use, and marijuana use by age category and sex. Models stratified by age category included sex, race/ethnicity, and past‐year major depressive episode as covariates. Models stratified by sex included age category, race/ethnicity, and past‐year major depressive episode as covariates. Results are presented as adjusted odds ratios (aOR) with 95% confidence intervals (95% CI). We used multiple‐imputed variables provided by NSDUH to limit the amount of missing data. Analyses were weighted to handle the NSDUH's complex sampling design and nonresponse using the “svy” command in Stata (version 18).

## RESULTS

Demographic statistics for the sample (stratified by sex at birth) are presented in Table 1. Across the total sample, the past‐year prevalence of past‐year substance use was 4.73% for tobacco (5.14% for males, 4.23% for females), 11.2% for marijuana (9.75% for males, 12.7% for females), and 17.2% for alcohol (15.0% for males, 19.5% for females). Substance use prevalence increased with age. Among 12–13‐year‐olds, past‐year tobacco, marijuana, and alcohol use were 1.04%, 1.90%, and 4.78%, respectively. Among 14–15‐year‐olds, prevalence increased to 4.69%, 11.2%, and 15.3%, respectively, and among 16–17‐year‐olds, past‐year use was 7.91% for tobacco, 19.1% for marijuana, and 29.7% for alcohol. Bivariate comparisons between sexual identity and substance use stratified by sex are presented in Table 2.

**TABLE 1:** Prevalence estimates with 95% confidence intervals for adolescents (ages 12–17 years) in the 2023 NSDUH data set stratified by sex (unweighted *N* = 10,361).

Variable	Male [95% CI]	Female [95% CI]
Age		
12–13‐Year Olds	31.2 [29.2, 33.3]	28.0 [26.0, 30.0]
14–15‐Year Olds	35.6 [33.5, 37.8]	35.6 [33.5, 37.8]
16–17‐Year Olds	33.1 [31.1, 35.2]	36.4 [34.2, 38.6]
Race/Ethnicity		
Non‐Hispanic White	50.2 [48.0, 52.4]	49.9 [47.6, 52.1]
Non‐Hispanic Black/African American	13.6 [12.2, 15.0]	13.9 [12.5, 15.4]
Non‐Hispanic Native American/Alaskan Native	0.86 [0.68, 1.07]	0.61 [0.40, 0.94]
Non‐Hispanic Native Hawaiian/Other Pacific Islander	0.20 [0.11, 0.35]	0.29 [0.10, 0.84]
Non‐Hispanic Asian	5.60 [4.68, 6.68]	5.42 [4.48, 6.53]
Non‐Hispanic More than One Race	3.52 [2.97, 4.17]	3.57 [2.94, 4.34]
Hispanic	26.1 [24.0, 28.2]	26.3 [24.2, 28.5]
Sexual identity		
Heterosexual	90.7 [89.5, 91.8]	62.4 [60.2, 64.6]
Gay/Lesbian	1.16 [0.81, 1.66]	4.50 [3.67, 5.51]
Bisexual	3.34 [2.74, 4.07]	16.3 [14.7, 18.0]
I use a Different term	1.67 [1.22, 2.30]	5.59 [4.67, 6.69]
Not Sure	3.11 [2.47, 3.91]	11.2 [9.76, 12.8]
Tobacco Use (Yes)	5.14 [4.26, 6.19]	4.23 [3.55, 5.20]
Alcohol Use (Yes)	15.0 [13.5, 16.6]	19.5 [17.8, 21.2]
Marijuana Use (Yes)	9.75 [8.58, 11.1]	12.7 [11.4, 14.2]
Past‐Year Major Depressive Episode (Yes)	8.97 [7.82, 10.3]	28.2 [26.2, 30.3]

**TABLE 2:** Bivariate comparisons between sexual identity for tobacco, alcohol, and marijuana.

	Tobacco use (No)	Tobacco use (Yes)	Alcohol use (No)	Alcohol use (Yes)	Marijuana use (No)	Marijuana use (Yes)
Males						
Heterosexual	95.1 [94.1, 96.0]	4.85 [3.95, 5.94]*	85.4 [83.7, 87.0]	14.6 [13.0, 16.3]	91.0 [89.6, 92.1]	9.00 [7.81, 10.4]***
Gay	95.7 [86.7, 98.7]	4.32 [1.32, 13.3]	75.2 [58.5, 86.7]	24.8 [13.3, 41.5]	80.7 [64.3, 90.6]	19.3 [9.35, 35.7]
Bisexual	86.8 [77.0, 92.8]	13.2 [7.16, 23.0]	78.8 [70.5, 85.2]	21.2 [14.7, 29.5]	83.7 [76.0, 89.3]	16.3 [10.7, 24.0]
Use a Different Term	93.0 [70.9, 98.6]	7.00 [1.36, 29.1]	75.9 [59.4, 87.1]	24.1 [12.9, 40.6]	74.2 [56.4, 86.5]	25.8 [13.5, 43.6]
Not Sure	95.9 [91.4, 98.1]	4.13 [1.93, 8.62]	87.0 [76.6, 93.1]	13.0 [6.86, 23.4]	87.7 [77.7, 93.6]	12.3 [6.35, 22.3]
Females						
Heterosexual	97.2 [96.3, 97.9]	2.78 [2.08, 3.71]***	83.0 [80.8, 84.9]	17.0 [15.1, 19.2]***	90.4 [88.6, 91.9]	9.63 [8.13, 11.4]***
Lesbian	98.0 [95.8, 99.0]	2.01 [0.95, 4.18]	68.5 [58.1, 77.4]	31.4 [22.6, 41.9]	82.4 [74.2, 88.5]	17.5 [11.5, 25.8]
Bisexual	91.5 [88.5, 95.0]	8.52 [6.28, 11.5]	74.1 [69.3, 78.4]	25.9 [21.6, 30.7]	78.3 [74.1, 82.1]	21.7 [18.0, 26.0]
Use a Different Term	91.2 [84.8, 95.0]	8.83 [4.97, 15.2]	78.8 [70.5, 85.3]	21.1 [14.7, 29.5]	84.5 [76.1, 90.3]	15.5 [9.68, 23.9]
Not Sure	94.7 [89.9, 97.3]	5.26 [2.69, 10.0]	82.1 [76.0, 82.2]	17.9 [13.1, 24.0]	86.4 [81.1, 90.4]	13.6 [9.58, 18.9]

*** *p* < .001,

* *p* < .05.

### Tobacco, marijuana, and alcohol by sexual identity

Tobacco use (*F*_11,10350_ = 6.04, *p* < .0001), alcohol use (*F*_11,10350_ = 15.0, *p* < .0001), and marijuana use (*F*_11,10350_ = 15.7, *p* < .0001) varied across sexual identities. Adjusting for covariates *F*_11,10350_ = 21.4, *p* < .0001), gay/lesbian youth had lower odds of tobacco use (aOR: 0.47, 95% CI: 0.24, 0.94) compared to heterosexual youth, but higher odds of alcohol use (aOR: 1.61, 95% CI: 1.06, 2.44). Bisexual youth also had greater odds of tobacco (aOR: 2.00, 95% CI: 1.37, 2.90), marijuana (AOR = 1.85, 95% CI: 1.43, 2.40), and alcohol (aOR: 1.32, 1.02, 1.69).

### Past year tobacco use by age

Logistic regression models stratified by age are presented in Table 3. Among 12–13‐year‐olds, gay/lesbian youth had significantly lower odds of tobacco use (AOR = 0.04, 95% CI: 0.005, 0.37), controlling for demographics (*F*_11,10350_ = 4.30, *p* < .0001). Among 12–13‐year‐olds (*F*_11,10350_ = 3.92), *p* < .0001), those who used marijuana (aOR: 9.67, 95% CI: 2.23, 41.8) or alcohol (aOR: 13.2, 95% CI: 2.56, 68.1) were at increased odds for using tobacco. Among 14–15 year olds, I *F*_11,10350_ = 4.01, *p* < .0001), there were no differences in use between sexual identities. Youth who used marijuana (aOR: 13.5, 95% CI: 6.63, 27.3) or alcohol (aOR: 3.56, 95% CI: 1.76, 7.21) had higher odds of tobacco use in the past year. Among 16–17 year olds (*F*_11,10350_ = 5.33, *p* < .0001), those who identified as Non‐Hispanic Black/African American (aOR: 0.38, 95% CI: 0.18, 0.79), female (aOR: 0.41, 95% CI: 0.27, 0.63), or gay/lesbian (aOR: 0.22, 95% CI: 0.0, 0.62) had lower odds of using tobacco in the past year. Compared to heterosexual youth, bisexual youth (aOR: 1.78, 95% CI: 1.11, 2.85) had higher odds of use. Moreover, youth who reported use of marijuana (aOR: 9.13, 95% CI: 5.50, 15.1) or alcohol (aOR: 4.66, 95% CI: 2.74, 7.92) had increased odds of tobacco use in the past year.

**TABLE 3:** Adjusted odds ratios (aORs) with 95% confidence intervals (95% CI) for tobacco, marijuana, and alcohol use across sexual orientation for respondents stratified by age category.

Variable	Tobacco use	Marijuana use	Alcohol use
12‐13‐year‐olds (*N* = 3082)			
Heterosexual	Ref	Ref	Ref
Gay/Lesbian	**0.04 [0.005, 0.37]**	0.92 [0.23, 3.63]	**3.71 [1.05, 13.10]**
Bisexual	2.78 [0.89, 8.73]	2.04 [0.66, 6.25]	1.24 [0.55, 2.80]
Other	2.83 [0.50, 15.9]	1.94 [0.37, 10.10]	0.61 [0.15, 2.55]
Not Sure	**0.29 [0.09, 0.97]**	1.49 [0.46, 4.83]	**0.23 [0.09, 0.62]**
14‐15‐year‐olds (*N* = 3718)			
Heterosexual	Ref	Ref	Ref
Gay/Lesbian	1.04 [0.40, 2.67]	1.18 [0.57, 2.44]	1.59 [0.83, 3.07]
Bisexual	1.79 [0.84, 3.80]	1.42 [0.89, 2.25]	1.29 [0.83, 2.01]
Other	2.21 [0.79, 6.21]	1.40 [0.63, 3.12]	0.89 [0.43, 1.85]
Not Sure	1.53 [0.61, 3.80]	1.69 [0.97, 2.93]	1.00 [0.56, 1.79]
16‐17‐year‐olds (*N* = 3561)			
Heterosexual	Ref	Ref	Ref
Gay/Lesbian	**0.22 [0.07, 0.62]**	1.49 [0.87, 2.57]	1.14 [0.66, 1.98]
Bisexual	**1.78 [1.11, 2.85]**	**1.91 [1.36, 2.69]**	1.14 [0.81, 1.61]
Other	1.18 [0.44, 3.17]	1.48 [0.77, 2.84]	1.17 [0.68, 2.03]
Not Sure	1.09 [0.45, 2.64]	1.09 [0.65, 1.85]	1.10 [0.67, 1.79]

*Note*: Models controlled for biological sex, race/ethnicity, and past‐year major depressive episode. Bold values indicate statistically significant results (*p* < .05).

### Past year marijuana use by age

Adjusted models examining 12–13‐year olds indicated no significant differences between sexual identities, compared to heterosexual youth (F_11,10350_ = 0.93, *p* = .99). Among 14–15‐year olds (*F*_11,10350_ = 7.10, *p* < .0001), youth who identified as Black/African American (aOR: 2.07, 95% CI: 1.22, 3.51), used tobacco (aOR: 13.1, 95% CI: 6.37, 26.9) or alcohol (aOR: 11.9, 95% CI: 8.00, 17.8) were at increased odds of marijuana use; no significant differences were found between identities. For 16–17‐year olds (*F*_11,10350_ = 11.3, *p* < .0001), youth who identified as Non‐Hispanic Black/African American (aOR: 2.52, 95% CI: 1.63, 3.87), Non‐Hispanic Native American (aOR: 3.47, 1.63, 7.39), used tobacco (aOR: 8.86, 95% CI: 5.33, 14.7), alcohol (aOR: 8.35, 95% CI: 6.02, 11.6), and who reported past‐year depression (aOR: 1.87, 95% CI: 1.31, 2.67) were at increased odds of marijuana. Compared to heterosexual youth, bisexual youth (aOR: 1.91, 95% CI: 1.36, 2.69) were at increased risk for marijuana use in the past year (Table 3).

### Past year alcohol use by age

Among 12–13‐year olds (*F*_11,10350_ = 5.13, *p* < .0001), compared to heterosexual youth, gay/lesbian youth had increased odds (aOR: 3.71, 95% CI: 1.05, 13.1) of engaging in alcohol use while those who identified as ‘not sure’ were at lower odds (aOR: 0.23, 95% CI: 0.09, 0.62) adjusting for covariates. Youth who used tobacco (aOR: 13.3, 95% CI: 2.60, 68.3), marijuana (aOR: 13.6, 95% CI: 5.66, 32.9), or reported depression in the past year (aOR: 3.21, 95% CI: 1.55, 6.66) were at increased odds of alcohol use in the past year. For 14‐15‐year‐olds (*F*_11,10350_ = 9.88, *p* < .0001), compared to Non‐Hispanic White youth, Non‐Hispanic Black/African American (aOR: 0.44, 95% CI: 0.27, 0.72), Asian (aOR: 0.25, 95% CI: 0.10, 0.60), and Hispanic (aOR: 0.61, 95% CI: 0.38, 0.99) were at lower odds of engaging in past‐year alcohol use. No differences between sexual identities were found. Youth who reported past‐year tobacco use (aOR: 3.50, 95% CI: 1.73, 7.10), marijuana use (aOR: 12.00, 95% CI: 8.04, 17.9), or major depression in the past year (aOR: 1.89, 95% CI: 1.24, 2.88) were at increased odds of alcohol use in the past year. Finally, for 16–17‐year‐olds (*F*_11,10350_ = 14.2, *p* < .0001), no differences were found between sexual identities. Compared to Non‐Hispanic White youth, Black/African American (aOR: 0.30, 95% CI: 0.18, 0.49) and Non‐Hispanic Native Alaskan/Pacific Islander (aOR: 0.01, 95% CI: 0.0001, 0.07) were at lower odds of alcohol use. Youth who reported past year tobacco use (aOR: 4.69, 95% CI: 2.74, 8.00), marijuana use (aOR: 8.34, 95% CI: 6.01, 11.6), or depression (aOR: 1.58, 95% CI: 1.15, 2.17) were at increased odds for alcohol use in the past year (Table 3).

### Past year substance use by biological sex

When stratified by sex (Table 4) (*F*_11,10350_ = 34.2, *p* < .0001), bisexual females had significantly higher odds of tobacco (aOR = 2.68, 95% CI: 1.67, 4.31) and marijuana use (aOR = 1.96, 95% CI: 1.43, 2.70), adjusting for covariates. Additionally, adjusting for covariates, youth identifying as “other” had elevated odds of marijuana use among males (aOR = 2.59, 95% CI: 1.14, 5.89) and tobacco use among females (aOR = 2.67, 95% CI: 1.26, 5.66).

**TABLE 4:** Adjusted odds ratios (aORs) with 95% confidence intervals (95% CI) for tobacco, marijuana, and alcohol use across sexual orientation for respondents stratified by self‐reported sex at birth.

Variable	Tobacco use	Marijuana use	Alcohol use
Males (*N* = 5342)			
Heterosexual	Ref	Ref	Ref
Gay	0.71 [0.21, 2.48]	1.92 [0.84, 4.41]	1.57 [0.71, 3.46]
Bisexual	**2.43 [1.16, 5.09]**	1.57 [0.96, 2.59]	1.23 [0.77, 1.97]
Other	1.05 [0.21, 5.27]	**2.59 [1.14, 5.89]**	1.30 [0.63, 2.66]
Not Sure	0.70 [0.30, 1.59]	1.18 [0.60, 2.33]	0.68 [0.34, 1.39]
Females (*N* = 5019)			
Heterosexual	Ref	Ref	Ref
Lesbian	0.55 [0.24, 1.27]	1.37 [0.83, 2.25]	1.59 [0.98, 2.60]
Bisexual	**2.68 [1.67, 4.31]**	**1.96 [1.43, 2.70]**	1.29 [0.96, 1.75]
Other	**2.67 [1.26, 5.66]**	1.19 [0.67, 2.12]	0.88 [0.54, 1.44]
Not Sure	1.73 [0.82, 3.68]	1.26 [0.83, 1.92]	0.89 [0.60, 1.31]

*Note*: Models controlled for race/ethnicity and past‐year major depressive episode. Bold values indicate statistically significant results (*p* < .05).

## DISCUSSION

The findings of this study highlight disparities in substance use among youth based on sexual orientation, with some evidence of elevated risk among bisexual adolescents. These results are generally consistent with prior research indicating that both distal and proximal minority stressors—such as discrimination, stigma, and identity‐based exclusion—can accumulate over time and contribute to increased substance use in adolescence.^10^, ^21^, ^22^ However, these disparities were not uniformly observed across all sexual minority subgroups or developmental stages. Patterns of disparity differed across sexual orientation, substance type, and developmental stage rather than uniformly escalating with age. For bisexual youth, disparities in tobacco and marijuana use became more pronounced in late adolescence (16–17 years). In contrast, gay/lesbian youth exhibited the strongest disparity in alcohol use at ages 12–13, while for tobacco use, heterosexual youth reported higher prevalence in early and mid‐adolescence. These findings suggest that substance use disparities among sexual minority youth emerge at different ages and vary in direction and magnitude depending on the substance, highlighting the importance of considering both developmental stage and substance type when evaluating inequities.^3^ In models stratified by sex (not controlling for age), bisexual females demonstrated significantly higher odds of tobacco and marijuana use compared to their heterosexual peers, and bisexual males showed elevated odds of tobacco use. However, in models stratified by age and adjusted for sex, disparities among bisexual youth were only statistically significant for tobacco and marijuana use among 16–17‐year‐olds. Among younger adolescents, particularly those aged 12–13, the largest disparity in substance use was observed in alcohol use among gay and lesbian youth. This pattern highlights the need to consider how substance use disparities may emerge or shift based on both age and sexual orientation subgroup, with some differences potentially reflecting developmental timing of risk exposure.

Literature consistently shows higher rates of substance use for individuals who identify as bisexual, compared to those who identify as lesbian/gay or heterosexual.^23^ The increased risk for tobacco use among bisexual females aligns with previous findings that bisexual women report earlier cigarette use initiation, for example, compared to other sexual minority groups.^24^ In contrast, bisexual males demonstrated elevated odds of tobacco use but not alcohol or marijuana use, suggesting potential differences in coping mechanisms and risk factors across gender. The increased risk for alcohol use among bisexual females aligns with previous research indicating that bisexual women typically report greater levels of alcohol use and heavy drinking.^25^

The unique experiences of bisexual adolescents, often referred to as binegativity, may be exacerbating the disparities observed in the differences by sex and age. Bisexual youth can encounter negative attitudes from both heterosexual and gay/lesbian individuals, leading to uniquely elevated minority stress.^26^ This hostility can make it challenging for bisexual individuals to find a safe and supportive community, increasing their risk for psychological distress and unhealthy coping behaviors that include substance use. Future research examining unique stressors experienced by bisexual youth (e.g., binegativity, bisexual invisibility) is recommended.

Interestingly, we did not observe significant differences in substance use across sexual orientation for 14–15‐year‐olds. This may reflect a developmental transition period where identity awareness is increasing, but behavioral health disparities have not yet fully emerged. Alternatively, it may reflect variability in disclosure or misclassification of identity at this age. Prior research has found that bisexual college‐aged women experience disproportionately poorer mental health outcomes than both their lesbian and heterosexual counterparts, including higher levels of anxiety, depressive symptoms, anger, self‐injury, and suicidal ideation.^21^ This study extends these findings to younger bisexual females (16–17 years), showing that they also experience heightened substance use risks compared to their peers. The cumulative effects of binegativity and elevated minority stress may be placing bisexual adolescent females in a particularly vulnerable position as they progress through adolescence, underscoring the need for targeted interventions that address both structural stigma and mental health disparities.^27^

Research indicates that fostering identity‐safe environments through social support and inclusivity programs can help mitigate substance use risks among youth navigating identity uncertainty.^28^ School‐based interventions such as Gay‐Straight Alliances have been shown to reduce minority stress and lower substance use behaviors among sexual minority adolescents by fostering a sense of belonging and safety.^29^, ^30^ Additionally, family‐affirming interventions, such as the Family Acceptance Project, have been linked to reduced substance use and improved mental health outcomes in LGBTQ+ individuals by addressing familial rejection and promoting supportive relationships.^31^, ^32^

Youth identifying as “other” or “not sure” about their sexual orientation also exhibited substance use disparities, though findings were more variable. Previous research suggests that the absence of a “something else” response option in national surveys may lead respondents to select categories that do not fully reflect their identities, potentially impacting health estimates.^33^ These findings underscore the need for further investigation into the unique experiences of youth in these categories and the role of support networks in promoting resilience and reducing substance use risk. Future research is warranted into how to better capture a wider and more inclusive range of sexual identities in surveillance surveys to investigate the nuances of complex sexual orientations and associated risks for substance use and mental health outcomes.

These findings have important public health implications. Prevention and intervention efforts should incorporate age‐specific and gender‐sensitive approaches to address the unique stressors faced by sexual minority youth. School‐based programs that foster supportive environments, promote mental health resources, and reduce stigma may be particularly effective in mitigating substance use risk.^34^ Additionally, ensuring access to identity‐affirming health care and peer support networks may help reduce substance use among youth who identify as “other” or are uncertain about their sexual orientation.^28^

This study was not without limitations. First, due to the cross‐sectional nature of the NSDUH data, we cannot establish any causal relationships. Second, the NSDUH does not assess gender identity (i.e., transgender, cisgender), and future research should examine differences by gender identity, as previous research has indicated that significant substance use disparities exist between these groups.^35^ Third, the NSDUH data is limited to civilian and noninstitutionalized populations, and results should not be generalized to groups not sampled. Finally, the omission of age adjustment in the sex‐stratified models likely improved model stability, but may have introduced residual confounding by age, leading to inflated effect sizes.

Future research should explore longitudinal trends in substance use disparities among sexual minority youth and examine the role of protective factors such as family support and community engagement. Further investigation is also needed to understand the specific experiences of youth who identify as “other” or “not sure,” as their substance use patterns were more variable. In addition, disaggregated analyses by both age and sex are critical for accurately identifying the timing and nature of disparities across subgroups.

## CONFLICT OF INTEREST STATEMENT

The authors declare no conflicts of interest. The authors alone are responsible for the content and writing of this paper.
